# Survey of challenges and best practice strategies for participant recruitment to research projects in northern ireland

**DOI:** 10.1186/1745-6215-14-S1-P85

**Published:** 2013-11-29

**Authors:** Lisa Maguire, Mike Clarke

**Affiliations:** 1 Queen's University Belfast, Belfast, UK

## 

Participant recruitment is understood to be one of the most difficult aspects of the research process. Researchers are now devoting increasing amounts of time and resources to understand how participants decide to take part in research and what researchers can do to make their work appeal to potential participants. The purpose of the study is to assess the problems experienced by researchers in Northern Ireland when recruiting human participants into trials and studies and to gain insight into how researchers handle and overcome these issues. The main research question being addressed by this research is to develop an understanding of the problems experienced by staff when recruiting human participants to research projects. Methods used to increase study recruitment were also examined. The participants in this research are investigators and other associated staff on research studies based in Northern Ireland. Potential participants were identified through contacts with research active organizations such as the academic researchers in Queen’s University Belfast and research physicians and clinical trialists employed by the Belfast Health and Social Care Trust. Each organization forwarded on the survey request via email or newsletters. Researchers willing to take part accessed the questionnaire through the Survey Monkey website. This study utilised a cross-sectional questionnaire design.

